# Association between obesity-associated markers and semen quality parameters and serum reproductive hormones in Chinese infertile men

**DOI:** 10.1186/s12958-020-00652-6

**Published:** 2020-09-29

**Authors:** Jian-Xiong Ma, Bin Wang, Hai-Song Li, Xue-Juan Jiang, Jia Yu, Cai-Fei Ding, Wang-Qiang Chen

**Affiliations:** 1 The Second Clinical Medical College, Zhejiang Chinese Medical University, Hangzhou, 310053 China; 2 Department of Reproductive Medicine, Zhejiang Provincial Integrated Chinese and Western Medicine Hospital, 208 East HuanCheng Road, Hangzhou, 310003 China; 3 Department of Andrology, Dongzhimen Hospital, Beijing University of Chinese Medicine, Beijing, 100007 China

**Keywords:** Body mass index (BMI), Semen parameters, Serum reproductive hormones, Serum leptin, Seminal leptin

## Abstract

**Background:**

The current evidence on the association between obesity-associated markers and semen quality, serum reproductive hormones and lipids remains inconsistent. In this study, we tested the hypothesis that, in infertile Chinese men, body mass index (BMI) negatively correlates with sperm concentration, serum total testosterone (TT), and high-density lipoprotein cholesterol (HDL-C). The relationship between other obesity-associated markers and semen quality parameters, serum reproductive hormones, lipids and leptin were also investigated.

**Methods:**

181 Chinese infertile men were recruited from September 2018 to September 2019. Their obesity-associated markers, semen parameters, and serum reproductive hormones, lipids and leptin were detected. Statistical analysis was performed to assess the relationship between obesity-associated markers and semen quality, serum reproductive hormones, lipids and leptin.

**Result(s):**

Statistically negative correlation was found between other obesity-associated markers (e.g. waist-to-hip ratio and waist-to-height ratio) and semen parameters (e.g. sperm concentration, ratio of progressive motility and ratio of non-progressive motility), while no significant correlation was found between BMI and semen quality, serum reproductive hormones, lipids and leptin. Ratio of morphologically normal sperm was negatively correlated with serum lipids including total cholesterol (TC) and low-density lipoprotein cholesterol (LDL-C), leptin and seminal superoxide dismutase. Ratio of progressive sperm, sperm concentration and ratio of morphologically normal sperm exhibited significantly lower values in overweight group than normal group. Estradiol (E2) and E2/TT were significantly higher in obese group than normal group, while TT level was significantly lower in obese group than normal group. Univariate and multivariate analysis indicated that TC was significantly associated with BMI. Serum leptin concentration was positively correlated with seminal leptin concentration in overweight and obese groups.

**Conclusion(s):**

No significant correlation was found between BMI and sperm concentration, serum TT and HDL-C, while other obesity-associated markers were found to negatively correlate with sperm concentration, ratio of progressive motility and ratio of non-progressive motility. Statistically significant correlations between serum reproductive hormones, lipids and leptin also existed in Chinese infertile men.

## Introduction

Overweight and obesity have been an important public health concern all over the world. Body mass index (BMI) is a typical indicator to measure health status of body weight, with 25–29.9 kg/m^2^ classified as “overweight” and ≥ 30 kg/m^2^ as “obese”. According to the World Health Organization (WHO), 39% of men ≥18 years old were overweight in 2016. Increased body weight has been associated with a higher frequency of some chronic diseases including metabolic disorders and multiple malignancies [1]. The markers used to reflect obesity include not only BMI, but also waist circumference (WC), waist-to-hip ratio (WHR) and waist-to-height ratio (WHtR). All these obesity-associated markers have also been used in clinical and epidemiological studies [2–5]. Infertility or subfertility is defined as the “disease characterized by the failure to establish a clinical pregnancy after 12 months of regular, unprotected, sexual intercourse” [6]. Infertility is considered as a disease of the reproductive system by WHO [7], which has affected at least 10% of population in developed countries [8].

Studies have suggested that male overweight and obese is significantly associated with infertility [9–11], but the results remain inconsistent. One study showed that no association was found between BMI and semen parameters [8]. A cohort study of 10,655 men in France showed that increase of BMI was significantly associated with the unfavorable seminal parameters [12]. However, in a cohort study of in 1231 infertile Chinese male populations, no obesity-associated markers were found to significantly associate with any of semen parameters [13]. Moreover, evidence on the association between underweight and semen quality is still unclear. Hormonal changes attributed to obesity can significantly affect the male reproductive function. These changes may include decreased levels of serum testosterone and gonadotropin, decreased binding capacity of sex hormone-binding globulin and increased serum oestradiol [14, 15]. Such alterations propose that endocrine dysregulation in obese males may be associated with more risk of unhealthy semen quality [16]. However, the current evidence on the association between BMI and serum reproductive hormones is limited and inconclusive, and further studies are needed.

Leptin is a 16 kDa secretory protein [17], which influences reproductive functions by stimulation of the gonadal functions in men [18, 19]. Seminal leptin concentrations display only a fraction of serum leptin levels [20]. It was demonstrated that seminal leptin concentrations were directly correlated with serum leptin concentrations [20]. So far, there have been limited studies about the relationship between obesity-associated markers and leptin concentration male infertile population.

The current evidence on the relationship between obesity and infertile in male population varies among different studies and different cohorts. Studies about obesity and infertile in Chinese male population has been rarely reported. In this study, we recruited 181 Chinese infertile men from September 2018 to September 2019. We aim to test the hypothesis that BMI negatively correlates with sperm concentration, serum total testosterone (TT), and serum high-density lipoprotein cholesterol (HDL-C) in infertile Chinese men. We also investigated the relationship between other obesity-associated markers and semen quality parameters, serum reproductive hormones, lipids and leptin.

## Materials and methods

### Participants

From September 2018 to September 2019, volunteer men were recruited consecutively from Department of Reproductive Medicine, Zhejiang Provincial Integrated Chinese and Western Medicine Hospital. The inclusion criteria were as follows: the men had to be 22–50 years old; be born and raised in China; no interceptive therapy to lose weight; the men and his partner had regular intercourse history and had the plan to conceive in the past 1 year but failed to conceive with no contraceptive measures; no history of any diseases associated with overweight or obese (e.g. severe varicocele, cryptorchidism, tumors, male sterilization, testicular torsion etc.); no evidence of severe accessory gonadal inflammation; no history of exposure to factors that significantly affect fertility; no evidence of obstructive azoospermia. Also, the men can obtain semen samples through masturbation. Altogether, 181 men were included and delivered a semen sample for the present study. The height, weight and waist of each participant were measured after the questionnaire. The sample size was calculated based on the formula: *n =* Z_1- α/2_^2^SD^2^/d^2^ [21]. For sperm concentration, we estimated 5% of type I error and precision of 10 (10^6^/ml) of either side (more or less than mean sperm concentration) and standard deviation, based on previous study in Chinese population [13], is 60.39 (10^6^/ml) then formula for sample size calculation will be 140 subjects. As for serum TT, we estimated 5% of type I error and precision of 0.60 (nmol/L) of either side, and standard deviation is 4.06 (nmol/L) [22], then the sample size will be 176 subjects. We were successful in achieving a sample size of 181 patients for analysis. As for HDL-C, we estimated 5% of type I error and precision of 0.05 (mmol/L) of either side, and standard deviation is 0.26 (mmol/L) [22], then the sample size will be 104 subjects.

This study was conducted in conformity with the Helsinki Declaration II and approved by the Ethics Committee of Zhejiang Provincial Integrated Chinese and Western Medicine Hospital. Written informed consent was given to all subjects before their inclusion.

### Semen analysis

Semen samples were collected via masturbation without the use of any lubricant after 2 to 7 days of abstinence. Participants produced the sample at the hospital in a room close to the laboratory. Semen analysis was performed in a sterile environment within 1 h of collection. The duration of abstinence was recorded, sperm concentration and motility of the fresh semen samples were primarily evaluated by conventional semen analysis according to the WHO laboratory manual [23]. An aliquot of semen sample was placed in a 20-μm-deep chamber slide (Leja Products BV, The Netherlands), and sperm motility including progressive motility (PR) and non-progressive motility (NP) was assessed using the Hamilton Thorne-TOX IVOS CASA system (Hamilton-Thorne Biosciences Inc., USA). Sperm concentration was also measured by the phase contrast microscope equipped with the analysis system. Microscope slides were prepared for sperm morphometry and morphology assessment. Sperm morphometry was conducted using the IVOS METRIX system (Hamilton-Thorne Biosciences Inc., USA) and sperm morphology was assessed on the prepared slides. The improved Papanicolaou staining method was applied to analyze sperm morphology [24]. Azoospermia was defined as zero sperm found on initial semen analysis, oligospermia included men with sperm present at concentrations < 15 million/mL, and normospermia was defined as sperm with concentrations ≥15 million/mL according to WHO guidelines [23]. Seminal superoxide dismutase (SOD) levels were determined by chemiluminescence assay using an automated C701 Immunoassay System (Roche, USA). After routine semen analysis, the semen was centrifuged, and the supernatant was stored at − 80 °C until analysis. Then the seminal supernatant was thawed, and the seminal leptin level was measured with a human leptin solid-phase sandwich ELISA using commercial kits (Abcam, #ab108879) according to the manufacturer’s instructions.

### Determination of serum reproductive hormones, lipids and leptin

A nonfasting blood sample was drawn the same day the semen sample was produced. Blood was centrifuged and serum was stored at − 80 °C until analysis. Sera were then thawed and analyzed for total testosterone (TT), luteinizing hormone (LH), follicle-stimulating hormone (FSH), estradiol (E2), prolactin (PRL) and progesterone (P). TT, LH, FSH, E2, PRL and P levels were determined by chemiluminescence assay using an automated Unicel Dxi 800 Access Immunoassay System (Beckman Coulter Inc., USA). Then the ratio of E2 and TT (E2/TT) was calculated. Serum lipids including total cholesterol (TC), triglycerides (TG), low-density lipoprotein cholesterol (LDL-C) and high-density lipoprotein cholesterol (HDL-C) levels were determined by chemiluminescence assay using an automated C701 Immunoassay System (Roche, USA). Serum leptin was measured with a human leptin solid-phase sandwich ELISA using commercial kits (Abcam #ab108879) according to the manufacturer’s instructions.

### Statistical analysis

BMI was calculated as weight (kg) divided by the square of height (m^2^). According the guidelines of WHO, BMI was categorized as underweight (< 18.5 kg/m^2^), normal weight (18.5–24.9 kg/m^2^), overweight (25–29.9 kg/m^2^) or obese (≥30 kg/m^2^) [25]. To test the robustness of our results, we also grouped BMI based on the Chinese criteria: underweight (< 18.5 kg/m^2^), normal weight (18.5–23.9 kg/m^2^), overweight (24–27.9 kg/m^2^) and obese (≥28 kg/m^2^) for sensitivity analyses [26]. Waist-to-hip ratio (WHR) was calculated as the ratio of waist circumference (WC) over the hip circumference. Waist-to-height ratio (WHtR) was calculated as the ratio of waist circumference over height. Analysis of variance and chi-square Χ^2^ tests were used to compare continuous and categorical variables between study groups. Shapiro-Wilk normality test was used to evaluate whether analyzed parameters were normally distributed. If the parameter was consistent with normal distribution, correlations between and within obesity associated markers, semen parameters and reproductive hormone levels were examined by Pearson test. If the parameter was consistent with non-normal distribution, correlations were examined by Spearman’s rho test. The differences between two groups with different number of samples were analyzed by independent-samples t-test (normally distributed) or Wilcoxon rank-sum test (non-normally distributed). Linear regression analysis was performed to test the relationship between seminal leptin concentration and serum leptin concentration in different BMI group. We used univariable and multivariable Cox regression models to assess the effects of all significant semen parameters/ serum reproductive hormones and BMI groups. We also used univariable model to assess the effects of obesity associated markers on sperm concentrations. All the data analysis was performed with R (version 3.6.2).

## Results

### Characteristics of participants

A total of 181 infertile men were recruited. This sample was further characterized in Table 1. Based on the BMI classification by WHO, 4 men were underweight (2.2%), 103 were normal (56.9%), 54 were overweight (29.8%), and 20 were obese (11.0%) (Supplementary Table 1). Based on the Chinese BMI category, 4 men were underweight (2.2%), 81 were normal (44.8%), 52 were overweight (28.7%), and 44 were obese (24.3%) (Supplementary Table 2). Thus, there were more participants classified into obese by Chinese criteria than WHO (24.3% vs 11.0%). The Wilcoxon rank-sum test indicated that age had no significant difference between normal and non-normal group (e.g. underweight, overweight and obese) in both WHO and Chinese classification, while height, weight, waist, hip, WHR and WHtR were significantly different between normal and underweight/overweight/obese group of both WHO and Chinese criteria (Supplementary Table 1 and Supplementary Table 2).

**Table 1:** Characteristics of the subjects in this study

Characteristic	Mean ± SD	Median (range)
Age (years)	30.92 ± 4.86	30 (21–49)
Height (cm)	172.71 ± 5.37	173 (160–192)
Weight (kg)	74.60 ± 12.60	72 (50–114)
WC (cm)	90.62 ± 11.47	89 (60–124)
Hip (cm)	99.13 ± 7.88	98 (75–122)
WHR	0.91 ± 0.06	0.91 (0.79–1.07)
WHtR	0.53 ± 0.07	0.51 (0.34–0.72)
Semen volume (mL)	2.92 ± 7.67	2.3 (0.5–4)
Ratio of PR sperm (%)	37.02 ± 18.96	36.6 (0–80.6)
Ratio of PR + NP sperm (%)	56.69 ± 20.15	57.4 (6.3–96)
Sperm concentration (10^6^/ml)	47.83 ± 27.97	43.2 (3.6–118.4)
Ratio of morphologically normal sperm (%)	3.53 ± 1.90	3.4 (0.5–14.1)
FSH	4.53 ± 2.18	4 (1.31–17.08)
LH	3.38 ± 1.60	3 (0.7–9.7)
P	0.64 ± 0.31	0.6 (0.03–1.74)
E2	36.93 ± 13.62	35 (12–79)
PRL	9.31 ± 4.21	8.74 (2.48–36.58)
TT	3.66 ± 1.09	3.61 (1.29–8.71)
E2/TT	11.18 ± 6.03	9.87 (2.37–44.96)
Serum Leptin (μg/L)	13.46 ± 2.31	13.62 (7.63–17.88)
Seminal Leptin (μg/L)	13.99 ± 2.49	14.03 (8.94–18.80)
Seminal SOD (pg/ml)	210.85 ± 38.57	211.18 (137.82–283.67)
TC (mmol/L)	4.90 ± 1.08	4.86 (2.71–9.11)
LDL-C (mmol/L)	2.84 ± 0.94	2.77 (1.21–5.49)
HDL-C (mmol/L)	1.12 ± 0.27	1.12 (0.17–1.82)
TG (mmol/L)	2.34 ± 1.47	2.11 (0.26–13.14)

### Association between obesity-associated markers and semen parameters/serum reproductive hormones

We firstly examined whether there were statistically significant associations between obesity-associated markers and semen quality parameters, serum reproductive hormones and lipids. Generalized obesity and abdominal obesity were defined using WHO Asia Pacific guidelines with WC cut-off as ≥90 cm [27], WHR cut-off as ≥0.9 [28], and WHtR cut-off as 0.5 [29, 30]. Semen parameters and serum reproductive hormones were dichotomized based on WHO guidelines [31]. By WHO BMI classification, the Chi-square test revealed a significant association between BMI and ratio of PR sperm (*P* < 0.05) and TC (*P <* 0.05) by both WHO Chinese BMI classification (Table 2). We also found that there was a significant association between WHR and sperm concentration (*P <* 0.05) and TC (*P <* 0.005) (Table 2).

**Table 2:** Association between semen parameters, serum reproductive hormones, lipids and leptin and obesity associated markers based on the dichotomized analyses for BMI, WC, WHR and WHtR

Variables	Status	Number	WHO BMI		Chinese BMI		Waist (cm)		WHR		WHtR	
			Χ^2^	*P* value	Χ^2^	*P* value	Χ^2^	*P* value	Χ^2^	*P* value	Χ^2^	*P* value
Semen volume (mL)	< 1.5	15	1.916	0.590	1.507	0.681	1.641	0.200	0.190	0.663	1.623	0.203
	≥ 1.5	166										
**Ratio of PR sperm (%)**	< 32	74	10.423	**0.015**	11.879	**0.008**	1.726	0.189	0.621	0.431	0.433	0.511
	≥ 32	107										
Ratio of PR + NP sperm (%)	< 40	46	3.215	0.360	2.134	0.545	2.686	0.101	3.033	0.082	0.858	0.354
	≥ 40	145										
**Sperm concentration (10**^**6**^**/ml)**	< 15	19	4.590	0.204	6.371	0.095	2.843	0.092	4.841	**0.028**	2.011	0.156
	≥ 15	164										
Ratio of morphologically normal sperm (%)	< 4	109	0.386	0.943	0.662	0.882	0.000	1.000	0.000	1.000	1.088	0.297
	≥ 4	72										
E2	< 20	16	1.933	0.926	1.623	0.951	0.730	0.694	0.013	0.908	0.824	0.364
	≥ 20 & ≤ 40	100										
	> 40	65										
TT	< 1.75	1	1.405	0.966	1.863	0.932	0.939	0.625	1.441	0.487	1.606	0.448
	≥ 1.75 & ≤ 3.5	87										
	> 3.5	93										
PRL	< 13.13	157	0.814	0.846	1.731	0.630	1.847	0.174	1.310	0.252	0.643	0.423
	≥ 13.13	24										
Serum leptin (μg/L)	< 12	50	0.098	0.992	0.298	0.960	0.007	0.932	0.712	0.399	0.158	0.691
	≥ 12 & ≤ 15	131										
	> 15											
Seminal leptin (μg/L)	< 12	47	0.192	0.979	0.521	0.914	0.387	0.534	0.069	0.793	0.000	1.000
	≥ 12 & ≤ 15	134										
	> 15											
**TC (mmol/L)**	< 5.18	111	14.493	**0.025**	18.916	**0.004**	5.217	0.074	13.485	**0.001**	1.560	0.458
	≥ 5.18 & ≤ 6.19	40										
	> 6.2	18										
LDL-C (mmol/L)	< 3.37	132	3.079	0.799	8.068	0.233	0.170	0.919	1.365	0.505	0.680	0.712
	≥3.37 & ≤ 4.12	22										
	> 4.14	16										
HDL-C (mmol/L)	< 1.04	64	3.273	0.774	5.822	0.444	2.671	0.263	4.756	0.093	0.661	0.718
	≥1.04 & < 1.55	94										
	≥1.55	12										
TG (mmol/L)	< 1.70	69	0.866	0.834	4.518	0.211	0.092	0.761	0.021	0.884	0.000	1.000
	≥1.70	101										

* *p <* 0.05; ** *p* < 0.01; *** *p* < 0.005; **** *p* < 0.0001; *WC* waist circumference, *WHR* waist-to-hip ratio, *WHtR* waist-to-height ratio

### Correlations of obesity-associated markers and the semen parameters

We performed Pearson test or Spearman’s rho test to evaluate the correlation between obesity-associated markers and semen parameters. Firstly, we found that statistically positive correlation existed between age and obesity-associated markers including WC, WHR and WHtR (Table 3). Secondly, we found that there were significant correlations among obesity-associated markers (Table 3). Specifically, weight had a strong positive correlation with other obesity associated markers including WC (coefficient: 0.887; *p <* 0.0001), hip (coefficient: 0.861; *p <* 0.0001), WHR (coefficient: 0.659; *p <* 0.0001) and WHtR (coefficient: 0.780; *P <* 0.0001) (Table 3). WHtR had a strong positive correlation with other obesity-associated markers including WC (coefficient: 0.960; *p <* 0.0001), hip (coefficient: 0.814; *p <* 0.0001) and WHR (coefficient: 0.859; *p <* 0.0001) (Table 3). Thirdly, we found that there was a significant correlation between obesity-associated markers and semen parameters. Specifically, sperm concentration was negatively correlated with WC, hip and WHtR (Table 3). Ratio of PR + NP sperm was negatively correlated with WHR and WHtR (Table 3). Fourthly, we found that there was significant correlation within semen parameters. Specifically, ratio of PR sperm had a strongly positive correlation with ratio of PR + NP sperm (coefficient: 0.954; *p <* 0.0001) (Table 3). Sperm concentration was positively correlated with ratio of PR sperm (coefficient: 0.385; *p <* 0.005) and ratio of PR + NP sperm (coefficient: 0.489; *p <* 0.005) (Table 3). No significant correlation was found between obesity-associated markers and ratio of morphologically normal sperm and semen volume.

**Table 3:** Correlation coefficients for relationships between age, obesity-associated markers and semen parameters

	Age	Height#	Weight	BMI	WC	Hip	WHR	WHtR#	Semen volume	PR	PR + NP	Sperm concentration
Height (cm)#	−0.085											
Weight (kg)	0.079	0.363****										
BMI	0.142	−0.005	0.926****									
WC (cm)	0.174*	0.136	0.887****	0.912****								
Hip (cm)	0.087	0.153*	0.861****	0.880****	0.897****							
WHR	0.234***	0.098	0.659****	0.686****	0.844****	0.523****						
WHtR#	0.223***	−0.115	0.780****	0.895****	0.960****	0.814****	0.859****					
Semen volume (mL)	−0.153*	0.124	−0.080	− 0.132	− 0.092	− 0.096	−0.049	− 0.077				
Ratio of PR sperm (%)	−0.121	− 0.004	− 0.031	− 0.035	− 0.098	−0.024	− 0.144	− 0.128	− 0.078			
Ratio of PR + NP sperm (%)	−0.109	0.001	−0.035	− 0.037	− 0.111	−0.037	− 0.156*	−0.148*	− 0.092	0.954****		
Sperm concentration (10^6^/ml)	−0.089	− 0.095	− 0.133	− 0.121	− 0.181*	− 0.170*	− 0.144	− 0.200**	− 0.081	0.385****	0.489****	
Ratio of morphologically normal sperm	−0.048	− 0.052	0.061	0.082	0.091	0.073	0.089	0.095	0.004	0.009	−0.017	− 0.017

* *p* < 0.05; ** *p* < 0.01; *** *p* < 0.005; **** *p* < 0.0001; # not normally distributed by Shapiro-Wilk normality test

### Correlations of obesity-associated markers and serum reproductive hormones, lipids and leptin

We evaluated the correlations between obesity-associated markers and serum reproductive hormones, lipids and leptin. Firstly, age was negatively correlated with LH and positively correlated with HDL-CH (Table 4). Secondly, significant correlations existed among serum reproductive hormones. Specifically, LH was positively correlated with FSH (coefficient: 0.417; *p* < 0.0001) and PRL (0.212; *p <* 0.005). P was significantly correlated with PRL and TT. PRL was positively correlated with TT but negatively correlated with E2/TT. Thirdly, significant correlations existed among serum lipids. Specifically, TC was significantly correlated with LDL-C (0.853; *p <* 0.0001), HDL-C (0.241; *p <* 0.005) and TG (0.443; *p <* 0.0001). TG was positively correlated with LDL-C but negatively correlated with HDL-C (Table 4). Fourthly, significant correlations existed between serum reproductive hormones and lipids. FSH was found to positively correlate with LDL-C and HDL-C. LH positively correlates with TG. Finally, serum leptin was strongly correlated with seminal leptin (0.701; *p <* 0.0001). Serum leptin was aslo correlated with seminal SOD, TC, E2, E2/TT and LDL-C. Seminal leptin was positively correlated with seminal SOD and serum lipids (e.g. TC, LDL-C and TG). Seminal SOD was positively correlated with LDL-C (Table 4).

**Table 4:** Correlation coefficients for relationships between age, obesity-associated markers, serum reproductive hormone, lipid and leptin levels

	Age	Height#	Weight	BMI	WC	Hip	WHR	WHtR#	FSH	LH	P	E2	PRL	TT	E2/TT	Serum leptin	Seminal leptin	Seminal SOD	TC	LDL-C	HDL-C#
FSH	− 0.046	− 0.052	0.013	0.033	0.039	0.012	0.057	0.130													
LH	−0.210**	−0.082	−0.025	0.005	− 0.014	0.008	−0.037	0.031	0.417****												
P	0.018	−0.058	0.018	0.038	0.032	0.035	0.019	0.002	−0.062	0.023											
E2	0.046	0.0239	0.041	0.011	−0.010	−0.012	−0.007	−0.030	0.014	0.022	−0.017										
PRL	−0.079	0.022	0.011	0.015	0.027	0.004	0.043	0.020	0.105	0.212***	0.167*	−0.149*									
TT	−0.106	0.025	0.032	0.034	0.011	−0.023	0.048	−0.004	0.034	0.105	0.222***	−0.050	0.170*								
E2/TT	0.081	0.016	0.015	0.016	−0.006	0.025	−0.044	−0.016	− 0.044	−0.048	− 0.112	0.726****	− 0.159*	−0.607****							
Serum leptin	0.127	0.0003	−0.049	−0.039	−0.049	− 0.021	−0.006	− 0.005	0.130	0.045	−0.109	0.200**	0.112	−0.148*	0.224***						
Seminal leptin	0.112	−0.012	−0.019	−0.007	0.032	0.026	0.034	0.032	0.119	0.066	−0.192**	0.190*	−0.038	−0.211**	0.263****	0.701****					
Seminal SOD	−0.021	0.089	−0.008	− 0.042	−0.028	− 0.026	−0.019	− 0.018	0.125	0.044	−0.080	0.117	−0.070	− 0.014	0.078	0.232***	0.287****				
TC	−0.069	− 0.006	0.038	0.043	0.040	0.057	0.014	−0.012	0.216***	0.111	−0.075	0.131	−0.056	−0.118	0.092	0.293****	0.263****	0.140			
LDL-C	−0.094	0.025	−0.022	− 0.007	−0.032	− 0.007	−0.047	− 0.034	0.187*	0.096	−0.097	0.137	−0.012	− 0.087	0.086	0.325****	0.294****	0.202**	0.853****		
HDL-C#	0.148*	0.065	−0.037	−0.082	−0.104	− 0.109	−0.028	− 0.125	0.060	− 0.064	0.059	− 0.074	−0.004	0.169*	−0.142	− 0.064	−0.058	− 0.107	0.241***	0.134	
TG	−0.109	−0.135	0.031	0.093	0.079	0.088	0.052	0.133	0.142	0.187*	0.022	0.125	−0.059	−0.170*	0.164*	0.140	0.166*	0.106	0.443****	0.249****	−0.294****

***WHR*** **waist-to-hip ratio,** ***WHtR*** **waist-to-height ratio**

### Comparisons of semen parameters, serum reproductive hormone, lipid and leptin levels based on the dichotomized analyses of BMI, WC, WHR and WHtR

We analyzed the association between BMI and semen quality. By WHO BMI classification, independent-samples t-test or Wilcoxon rank-sum test indicated that ratio of PR + NP sperm (%) was significantly lower in underweight group than normal group (Table 5). Ratio of PR sperm (%), ratio of PR + NP sperm (%), sperm concentration (10^6^/ml) and ratio of morphologically normal sperm (%) were significantly lower in overweight group than normal group (Table 5). By Chinese BMI classification, only ratio of morphologically normal sperm (%) was found significantly lower in overweight/obese group than normal group (Supplementary Table 3). Taken together, we found significant association in semen parameters between different BMI groups but inconsistency existed by using WHO and Chinese BMI categories. For WC, we found that ratio of PR + NP sperm and sperm concentration were significantly lower in WC ≥ 90 cm than WC < 90 cm (Table 5). For WHR, we found that ratio of PR sperm was significantly lower in WHR ≥0.9 group than < 0.9 group (Table 5).

**Table 5:** Comparisons of serum reproductive hormone, lipids and leptin levels based on the dichotomized analyses for BMI, WC, WHR and WHtR

Variable	WHO BMI category				WC (cm)			WHR			WHtR		
	Underweight (*N* = 4)	Normal (*N* = 103)	Overweight (*N* = 54)	Obese (*N* = 20)	< 90 (*N* = 95)	≥ 90 (*N* = 86)	*P* value	< 0.9 (*N* = 76)	≥0.9 (*N* = 105)	*P* value	< 0.5 (*N* = 70)	≥0.5 (*N* = 111)	*P* value
Semen volume (mL)	2.53 ± 0.33^a^	2.35 ± 0.56	2.42 ± 0.69	2.16 ± 0.66	2.40 ± 0.52	2.30 ± 0.70	0.290	2.34 ± 0.55	2.37 ± 0.65	0.797	2.40 ± 0.53	2.33 ± 0.66	0.797
Ratio of PR sperm (%)	21.15 ± 6.55^a^	39.44 ± 18.50	33.47 ± 18.33* ^**a**^	37.30 ± 22.60 ^a^	39.09 ± 18.18	34.73 ± 19.64	0.124	40.22 ± 18.01	34.70 ± 19.29	**0.050**^**a**^	39.05 ± 18.41	35.74 ± 19.28	0.226^a^
Ratio of PR + NP sperm (%)	41.15 ± 8.97*^a^	59.37 ± 19.46	51.68 ± 20.39* ^**a**^	59.52 ± 21.72 ^a^	59.23 ± 19.19	53.88 ± 20.91	**0.063** ^**a**^	59.73 ± 18.24	54.49 ± 21.14	0.092^a^	59.25 ± 18.73	55.08 ± 20.92	0.159 ^a^
Sperm concentration (10^6^/ml)	67.13 ± 38.59^a^	50.91 ± 27.31	39.81 ± 23.97**	49.73 ± 35.41	52.22 ± 27.72	42.97 ± 27.61	**0.026**	51.49 ± 23.79	45.17 ± 30.34	0.119	52.07 ± 27.52	45.15 ± 28.05	0.104
Ratio of morphologically normal sperm (%)	4.05 ± 1.34^a^	3.86 ± 2.01	2.944 ± 1.44***	3.32 ± 2.14 ^a^	3.38 ± 1.64	3.71 ± 2.14	0.248	3.43 ± 1.73	3.61 ± 2.00	0.510	3.24 ± 1.68	3.72 ± 2.01	0.087
FSH	3.98 ± 1.53^a^	4.33 ± 2.11	4.86 ± 2.40	4.76 ± 2.00 ^a^	4.47 ± 2.38	4.59 ± 1.95	0.730	4.40 ± 2.32	4.62 ± 2.07	0.508	4.34 ± 2.43	4.65 ± 2.01	0.376
LH	3.13 ± 1.22 ^a^	3.46 ± 1.74	3.28 ± 1.53	3.30 ± 1.05 ^a^	3.39 ± 1.61	3.37 ± 1.60	0.960	3.40 ± 1.63	3.36 ± 1.57	0.872	3.26 ± 1.46	3.45 ± 1.68	0.415
P	0.69 ± 0.18 ^a^	0.68 ± 0.34	0.57 ± 0.26* ^**a**^	0.63 ± 0.26 ^a^	0.65 ± 0.30	0.63 ± 0.33	0.445^a^	0.65 ± 0.28	0.64 ± 0.33	0.816	0.64 ± 0.30	0.64 ± 0.32	0.978
E2	33.75 ± 10.69 ^a^	34.52 ± 12.91	38.22 ± 13.90 ^a^	46.5 ± 13.03**** ^**a**^	36.56 ± 13.53	37.34 ± 13.78	0.853^a^	37.46 ± 13.48	36.55 ± 13.70	0.657	38.34 ± 13.68	36.04 ± 13.57	0.271
PRL	10.52 ± 3.66 ^a^	9.63 ± 4.80	8.85 ± 3.52 ^a^	8.72 ± 2.30 ^a^	9.15 ± 3.76	9.49 ± 4.66	0.593	8.97 ± 3.37	9.56 ± 4.70	0.329	8.92 ± 3.48	9.56 ± 4.61	0.293
TT	5.32 ± 2.47 ^a^	3.84 ± 1.01	3.442 ± 0.93* ^**a**^	2.98 ± 0.98**** ^**a**^	3.64 ± 1.00	3.68 ± 1.19	0.794	3.68 ± 1.18	3.65 ± 1.02	0.945^a^	3.63 ± 1.01	3.68 ± 1.15	0.749
E2/TT	7.21 ± 3.55 ^a^	9.71 ± 4.72	12.26 ± 7.04*	16.64 ± 5.94**** ^**a**^	11.04 ± 6.14	11.34 ± 5.94	0.742	11.41 ± 6.60	11.01 ± 5.59	0.666	11.70 ± 6.62	10.85 ± 5.64	0.378
Serum Leptin (μg/L)	11.55 ± 1.14 ^a^	12.62 ± 2.10	14.89 ± 1.94**** ^**a**^	14.35 ± 2.27*** ^**a**^	13.45 ± 3.39	13.47 ± 2.24	0.889^a^	13.24 ± 2.41	13.62 ± 2.23	0.326^a^	13.48 ± 2.44	13.45 ± 2.24	0.837^a^
Seminal Leptin (μg/L)	12.85 ± 1.60 ^a^	13.04 ± 2.18	15.71 ± 2.07**** ^**a**^	14.44 ± 2.66* ^**a**^	13.87 ± 2.57	14.12 ± 2.41	0.487	13.81 ± 2.56	14.12 ± 2.44	0.423	13.99 ± 2.65	13.99 ± 2.40	0.964^a^
Seminal SOD (pg/ml)	214.77 ± 51.38 ^a^	207.0 ± 36.91	214.9 ± 41.06	218.7 ± 38.27 ^a^	209.31 ± 38.72	212.55 ± 38.55	0.573	211.49 ± 38.27	210.40 ± 38.77	0.848	211.36 ± 36.86	210.53 ± 39.77	0.903
TC (mmol/L)	4.28 ± 0.59 ^a^	4.53 ± 0.83	5.33 ± 1.17**** ^**a**^	5.76 ± 1.18**** ^**a**^	4.86 ± 0.97	4.94 ± 1.20	0.615	4.82 ± 0.98	4.96 ± 1.15	0.408	4.84 ± 1.01	4.94 ± 1.13	0.574
LDL-C (mmol/L)	2.44 ± 0.70	2.55 ± 0.66	3.042 ± 0.98*** ^**a**^	3.90 ± 1.25****	2.85 ± 0.93	2.84 ± 0.96	0.951	2.79 ± 0.91	2.88 ± 0.96	0.502	2.82 ± 0.91	2.86 ± 0.96	0.824
HDL-C (mmol/L)	1.43 ± 0.09* ^**a**^	1.15 ± 0.23	1.13 ± 0.27*** ^**a**^	0.88 ± 0.37 ^a^	1.13 ± 0.32	1.11 ± 0.22	0.389^a^	1.13 ± 0.33	1.11 ± 0.23	0.761^a^	1.14 ± 0.31	1.11 ± 0.25	0.318^a^
TG (mmol/L)	0.89 ± 0.32** ^**a**^	1.96 ± 1.38	2.91 ± 1.44****	3.00 ± 1.36**** ^**a**^	2.30 ± 1.65	2.38 ± 1.25	0.708	2.28 ± 1.72	2.38 ± 1.27	0.679	2.15 ± 1.16	2.45 ± 1.63	0.147

* *p* < 0.05; ** *p* < 0.01; *** *p* < 0.005; **** *p* < 0.0001; ^a^ Wilcoxon rank-sum test

We also analyzed the association between BMI and serum reproductive hormones, lipids and leptin. By WHO BMI classification, independent-samples t-test or Wilcoxon rank-sum test indicated that E2 was significantly higher (*p* < 0.0001), while TT was significantly lower (*p <* 0.0001), in obese group than normal (Table 5). Both serum and seminal leptin were significantly higher in overweight and obese group than normal group (Table 5). Lipids including TC, LDL-C and TG were significantly higher in overweight and obese group than normal group (Table 5). Same analysis was also performed based on Chinese BMI classification (Supplementary Table 3).

Collectively, we found that statistically significant differences of semen parameters, serum reproductive hormones, lipids and leptin existed regarding BMI groups but there were variations by using WHO and Chinese BMI categories. Only some of the semen parameters were found to have significant difference between different WC (e.g. ratio of PR + NP sperm and sperm concentration) and WHR (e.g. ratio of PR sperm) groups.

### Correlations of semen parameters and serum reproductive hormones, lipids and leptin

We also analyzed the correlations between semen parameters and serum reproductive hormones, lipids and leptin. We found that semen volume was negatively correlated with PRL, and ratio of morphologically normal sperm was negatively correlated with serum leptin, seminal leptin, seminal SOD, TC and LDL-C (Table 6). We also found that HDL-C was significantly correlated with ratio of PR sperm, ratio of PR + NP sperm and sperm concentrations (Table 6). Therefore, statistically significant correlations existed between semen parameters and serum reproductive hormones.

**Table 6:** Correlation coefficients for relationships between semen parameters, serum reproductive hormone, lipids and leptin

	FSH	LH	P	E2	PRL	T	E2/T	Serum Leptin	Seminal Leptin	Seminal SOD	TC	LDL-C	HDL-C#	TG
Semen volume (mL)	−0.069	0.068	−0.050	0.024	−0.165*	− 0.016	− 0.007	0.116	0.022	− 0.008	− 0.023	0.026	− 0.057	− 0.028
Ratio of PR sperm (%)	− 0.031	0.014	− 0.009	0.075	0.020	0.054	0.012	−0.036	−0.055	0.035	0.052	0.028	0.173*	−0.050
Ratio of PR + NP sperm (%)	0.019	0.069	0.022	0.067	0.045	0.045	0.012	−0.028	−0.057	0.006	0.075	0.044	0.190*	−0.039
Sperm concentration (10^6^/ml)	−0.042	0.001	0.102	0.016	0.088	−0.008	0.011	0.008	−0.017	−0.036	0.092	0.036	0.207**	0.012
Ratio of morphologically normal sperm (%)	−0.099	− 0.116	0.004	−0.013	0.075	0.179*	−0.115	−0.223**	− 0.201**	−0.243***	− 0.162*	−0.175*	− 0.022	−0.044

### Independent effects between obesity associated markers and semen parameters / serum reproductive hormones

For univariate Cox regression analysis of each semen parameters and serum reproductive hormones independently, we found that only TC was significantly associated with BMI by both WHO and Chinese classifications (Table 7 and Supplementary Table 4). For multivariate Cox regression analysis, we firstly considered only semen parameters (Model A, Table 7) and found that there was no significant association between semen parameters and BMI by both WHO and Chinese classifications (Table 7 and Supplementary Table 4). Secondly, we considered only serum reproductive hormones, lipids and leptin (Model B, Table 7) and found that there was a significant association between TC and BMI by WHO classification (Table 7), while there was no significant association was found between Chinese BMI and serum reproductive hormones, lipids and leptin (Supplementary Table 4). Thirdly, we considered all semen parameters, serum reproductive hormones, lipids and leptin and found that there was a significant association between TC and BMI by both WHO and Chinese classifications (Table 7 and Supplementary Table 4). We also found that seminal leptin was significantly associated with BMI by WHO classification (Table 7).

**Table 7:** Univariate and multivariate analysis of semen parameters, serum reproductive hormone, lipids and leptin regarding WHO BMI category (normal: 18.50–24.99 versus pathologic > 24.99)

Variable	Univariate analysis		Multivariate Model A		Multivariate Model B		Multivariate Model C	
	RR (95% CI)	*P* value	RR (95% CI)	*P* value	RR (95% CI)	*P* value	RR (95% CI)	*P* value
Semen volume (mL)	2.1 (0.96–4.8)	0.065	2.01 (0.87–4.6)	0.100	–	–	2.84 (0.76–10.6)	0.120
Ratio of PR sperm (%)	1.2 (0.75–1.9)	0.450	1.19 (0.66–2.10)	0.552	–	–	1.11 (0.50–2.5)	0.790
Ratio of PR + NP sperm (%)	0.99 (0.58–1.7)	0.960	0.70 (0.30–1.60)	0.398	–	–	0.72 (0.27–2.0)	0.523
Sperm concentration (10^6^/ml)	1.2 (0.65–2.2)	0.560	1.28 (0.54–3.00)	0.576	–	–	1.45 (0.51–4.1)	0.490
Ratio of morphologically normal sperm (%)	0.9 (0.56–1.4)	0.670	0.91 (0.57–1.50)	0.713	–	–	1.08 (0.56–2.1)	0.825
E2	1.6 (0.64–4.1)	0.310	–	–	0.49 (0.17–1.40)	0.180	0.57 (0.18–1.8)	0.337
PRL	0.98 (0.51–1.9)	0.940	–	–	1.17 (0.55–2.50)	0.681	1.30 (0.57–3.0)	0.531
Serum leptin (μg/L)	1.3 (0.76–2.2)	0.340	–	–	0.74 (0.37–1.50)	0.387	0.49 (0.20–1.2)	0.118
**Seminal leptin (μg/L)**	1.2 (0.72–2.1)	0.440	–	–	1.90 (0.95–3.8)	0.071	2.29 (1.02–5.1)	**0.044**
**TC (mmol/L)**		**0.035**						
< 5.18	1.00		–	–	1.00		1.00	
**≥ 5.18 & ≤ 6.19**	−0.12 (1.2–4.1)		–	–	3.46 (1.35–8.90)	**0.010**	4.99 (1.75–14.2)	**0.003**
> 6.2	−0.12 (0.46–1.7)		–	–	0.51 (0.11–2.40)	0.397	0.72 (0.14–3.6)	0.686
LDL-C (mmol/L)		0.930						
< 3.37	1.00		–	–	1.00		1.00	
≥ 3.37 & ≤ 4.12	−0.034 (0.56–2.3)		–	–	0.57 (0.20–1.60)	0.297	0.38 (0.11–1.3)	0.113
> 4.14	−0.034 (0.46–2)		–	–	1.35 (0.24–7.6)	0.730	1.56 (0.31–8.0)	0.592
HDL-C (mmol/L)		0.380						
< 1.04	1.00		–	–	1.00		1.00	
≥ 1.04 & < 1.55	0.69 (0.77–2.1)		–	–	1.55 (0.85–2.8)	0.153	1.26 (0.64–2.5)	0.504
≥ 1.55	0.69 (0.68–5.8)		–	–	3.01 (0.78–11.6)	0.109	4.17 (0.96–18.0)	0.056
TG (mmol/L)	0.91 (0.55–1.5)	0.700	–	–	0.84 (0.48–1.5)	0.534	0.79 (0.41–1.5)	0.463

We also performed univariate Cox regression analysis to evaluate the independent effect of obesity-associated markers on sperm concentrations. No significant association existed between sperm concentrations and BMI (Supplementary Table 5). Also, no significant association was found between sperm concentrations and other obesity-associated markers (Supplementary Table 5).

### Relationship between serum leptin and seminal leptin concentration

**Fig. 1:**
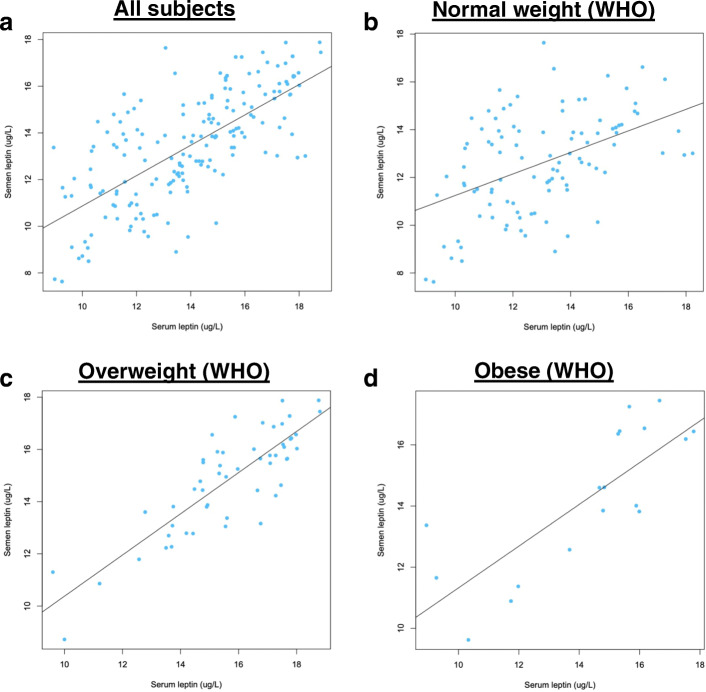
Correlation between serum leptin concentration and seminal leptin concentration by WHO BMI classifications. **a**, All subjects; **b**, Normal weight; **c**, Overweight; **d**, Obese. WHO, World Health Organization

**Fig. 2:**
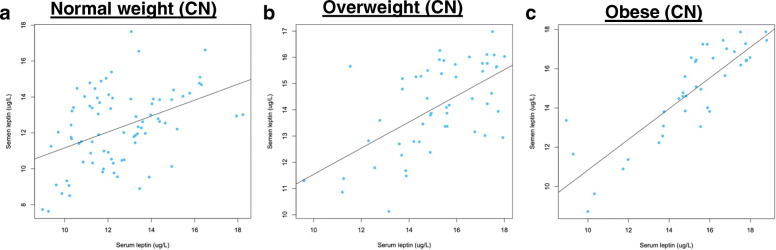
Correlation between serum leptin concentration and seminal leptin concentration by Chinese BMI classifications. **a**, Normal weight; **b**, Overweight; **c**, Obese. CN, Chinese

We found that serum leptin and seminal leptin concentrations were significantly higher in overweight and obese group than normal group. Then we performed linear regression analysis on serum leptin concentration and seminal leptin concentration in different BMI groups. In all participants, we found that serum leptin concentration was positively correlated with seminal leptin concentration (*R*^*2*^ = 0.49, *P* < 0.0001) (Fig. 1a). By WHO BMI classification, the correlation coefficient became weaker in normal BMI group (*R*^*2*^ = 0.22, *P <* 0.0001) (Fig. 1b), while the correlation coefficient became much stronger in overweight (*R*^*2*^ = 0.71, *P <* 0.0001) (Fig. 1c) and obese group (*R*^*2*^ = 0.64, *P <* 0.0001) (Fig. 1d). By Chinese BMI classification, we found that the correlation coefficient became much weaker in normal group (*R*^*2*^ = 0.18, *P <* 0.0001) (Fig. 2a) and overweight group (*R*^*2*^ = 0.36, *P <* 0.0001) (Fig. 2b), while the correlation coefficient became much stronger in obese group (*R*^*2*^ = 0.73, *P <* 0.0001) (Fig. 2c)**.** Collectively, we found that serum leptin concentration was positively correlated with seminal leptin concentration in overweight and obese cohorts, while correlation coefficients varied between WHO and Chinese BMI classification.

## Discussion

In this study, we analyzed the relationships between obesity-associated markers and semen parameters, serum reproductive hormones, lipids and leptin in 181 Chinese infertile men. We find that BMI has no significant correlation with semen quality parameters, while WC, hip and WHtR are negatively correlated with sperm concentration. We also find that there is no significant correlation between obesity-associated markers and serum reproductive hormones, lipids and leptin. Interestingly, we find that statistically significant correlations exist among semen parameters, serum reproductive hormones, lipids and leptin. Notably, ratio of PR sperm has a strongly positive correlation with ratio of PR + NP sperm. TC has a strongly positive correlation with LDL-C.

Our data showed that WC, WHR and WHtR were positively related to age, indicating that aging might lead to more serious obesity degree in adult Chinese men. Age was negatively related to semen volume and LH and positively related with HDL-C. But the correlation was not as strong as previous study in Chinese men [32], which might be explained by the smaller sample size in our study.

There have been many inconsistent studies about the association between BMI and semen quality parameters. Some studies indicated that higher BMI levels affect semen quality parameters including semen volume, motile sperm and sperm concentration [33–36], while many studies showed no association between BMI and semen quality parameters [37, 38]. In one recent cross-sectional study, based on a strict classification on weight and analysis of the effects of metabolically healthy obesity (MHO) on male fertility, Cazzaniga W et al. found that patients with metabolically unhealthy obesity (MUHO) were more frequently to have secondary and compensated hypogonadism compared with patients with metabolically healthy non-obese (MHNO) [39]. This study highlights the importance of weight control to patients with MUHO. Based on this classification, the authors found no significant effect of metabolic condition on semen parameters as compared with MHNO [39]. In our study, we made the classification based on BMI by both WHO and Chinese classifications. We also considered other obesity-associated markers in male infertility. We found that only ratio of PR sperm was significantly associated with BMI status, while no correlation existed between BMI and semen parameters. Interestingly, we found that WC, hip and WHtR had a negative correlation with sperm concentration, and WHR and WHtR had a negative correlation with ratio of PR + NP sperm. Our study highlighted the importance to take other obesity-associated markers into consideration. Moreover, we found that overweight group exhibited a significantly lower semen quality than normal group, including ratio of PR sperm, ratio of PR + NP sperm, sperm concentration and ratio of morphologically normal sperm, which was consistent with many previous studies [32, 33]. Interestingly, no significant statistical difference of semen parameters was found between obese and normal group. Higher BMI was found to have negative impact on semen quality [32], while we found that ratio of PR + NP sperm was significantly lower in underweight group than normal group. Therefore, our study suggests that men with overweight and underweight BMI tend to have the poorer semen quality than normal BMI.

Previous studies observed an inverse relationship between overweight/obesity and androgen levels [1, 40]. When taking metabolic condition into consideration, Cazzaniga W et al. found that MHO had lower TT but higher E2 circulating values [39]. In our study, we found that overweight and obese group exhibited a significantly lower level of TT and HDL-C than that in normal group, while higher level of P, E2, E2/TT, TC, LDL-C and TG than that in normal group. A previous study indicated that a TT/E2 decrease was associated with male infertility and sperm defect, but had no impact on sperm concentration and motility [41]. This implies that TT/E2 plays important roles in spermatogenesis and fertilization ability. Therefore, the study of TT/E2 has important implications in male reproductive health and infertility therapy. In this study, we observed significantly decreased ratio of normally morphological sperm in overweight group. Together with the same previous study, TT/E2 might be considered as a potential indicator of male fertility in overweight population.

We could not find any difference in FSH between different levels of each obesity-associated marker, which was consistent with previous study [8, 40]. FSH is secreted by pituitary and stimulate testis to produce sperm. FSH secreting will be enhanced by a negative feedback regulation when sperm count was downregulated. No significant difference of sperm count was found between overweight and normal group, so it was reasonable that no significant difference of FSH was observed. LH stimulates testicular stromal cells to produce testosterone. LH level will be upregulated by negative feedback loop, but its change is not as remarkable as FSH and sperm count. TT level was significantly lower in overweight and obese group than normal group, but still within normal scale (1.75–7.81), so it was possible that LH has no significant difference between overweight/obese and normal group.

We found that serum leptin and seminal leptin were significantly higher in overweight and obese groups than normal group. Leptin plays a key role in the regulation of body fat mass by regulating appetite and metabolism while balancing energy intake and expenditure. Previous studies have observed that a progressive increase in serum leptin concentration was positively associated with an increase in BMI [42, 43]. Seminal leptin concentrations display a fraction of serum leptin levels. In this study, we found that serum leptin was positively correlated with seminal leptin. This correlation became much stronger in overweight and obese group, highlighting the importance of BMI on leptin level.

The relationship between leptin and semen parameters remains unclear. One study showed that there is no correlation between seminal leptin level and semen quality parameters [44], while another study indicated that a pathophysiological relevance of seminal leptin in sperm motility existed [45]. In our study, we found that serum leptin and seminal leptin levels were inversely correlated with ratio of morphologically normal sperm. Previous study showed that leptin administration in adult rat lead to upregulation of serum FSH and LH, implying that leptin can possibly affect male infertility by hormone profile modulation [46]. In our study, we found that serum leptin and seminal leptin was positively correlated with E2, E2/TT, seminal SOD, TC and LDL-C. Our study showed that leptin concentration was inversely correlated with TT, which was consistent with previous study [47]. These results might imply that leptin level was associated with overweight level and the increase of seminal SOD.

Studies about the correlation between semen parameters and serum reproductive hormone have been rarely reported. In one study about Chinese male population, sperm concentration and morphology was found to inversely correlate with LH and FSH [13]. In this study, we found that HDL-C level was positively correlated with ratio of PR sperm, ratio of PR + NP sperm and sperm concentration, while ratio of morphologically normal sperm was inversely correlated with seminal SOD, TC and LDL-C. We also found that variations in semen parameters and serum reproductive hormones existed by WHO and Chinese BMI categories, which could be explained by the sample size, ethnicity, dietary and climate differences.

## Conclusions

Our study found that (1) statistically significant differences were found in semen parameters, serum reproductive hormones, lipids and leptin between different BMI groups; (2) statistically significant correlations exist between semen parameters and serum reproductive hormones, lipids and leptin. Therefore, our findings indicated that consideration of other obesity-associated markers (e.g. WC, Hip, WHR and WHtR) will provide more comprehensive evaluation on male infertility than BMI alone. Moreover, together with obesity-associated markers, serum reproductive hormones, lipids and leptin could also be included to evaluate male semen quality. However, limitations existed in our study. Compared with previous studies [1, 13, 39], our single center-based study recruited a relatively small, homogeneous, same-ethnicity cohort of infertile men, which might lead to selection biases. Therefore, further studies across multiple centers and different ethnicities are needed to corroborate our findings

## Supplementary information

**Additional file 1: Table S1.** Characteristics of the study subjects by WHO BMI category. **Table S2.** Characteristics of the study subjects by Chinese BMI category. **Table S3.** Characteristics of semen quality parameters, serum reproductive hormones, lipids and leptin by Chinese BMI category. **Table S4.** Univariate and multivariate analysis of semen parameters, serum reproductive hormone, lipids and leptin levels regarding Chinese BMI category (normal: 18.50–23.9 versus pathologic > 23.9). **Table S5.** Association between sperm concentration and obesity-associated markers.

## Data Availability

The data and materials are available from the corresponding author on reasonable requests.
