# Supplementary material for: Association between obesity-associated markers and semen quality parameters and serum reproductive hormones in Chinese infertile men
Source: Reprod Biol Endocrinol. 2020 Sep 29;18:95. doi: 10.1186/s12958-020-00652-6 (PMC7523062; doi:10.1186/s12958-020-00652-6)
Supplement: Supplementary file 1 — Additional file 1: Table S1. Characteristics of the study subjects by WHO BMI category. Table S2. Characteristics of the study subjects by Chinese BMI category. Table S3. Characteristics of semen quality parameters, serum reproductive hormones, lipids and leptin by Chinese BMI category. Table S4. Univariate and multivariate analysis of semen parameters, serum reproductive hormone, lipids and leptin levels regarding Chinese BMI category (normal: 18.50–23.9 versus pathologic > 23.9). Table S5. Association between sperm concentration and obesity-associated markers. [file 12958_2020_652_MOESM1_ESM.docx]

**Supplementary Table 1 Characteristics of the study subjects by WHO BMI category**

| Characteristic | Underweight (n=4) | | Normal (n=103) | | Overweight (n=54) | | Obese (n=20) | |
| --- | --- | --- | --- | --- | --- | --- | --- | --- |
|  | Mean ± SD | Median (range) | Mean ± SD | Median (range) | Mean ± SD | Median (range) | Mean ± SD | Median (range) |
| Age (years) | 27 ± 2.16 | 27.5 (24-29) | 30.45 ± 4.43 | 30 (22-49) | 32.05 ± 5.41 | 31 (21-49) | 31.05 ± 5.28 | 30 (23-41) |
| Height (cm) | 172.5 ± 4.93 | 172.5 (167-178) | 172.38 ± 5.12 | 172 (160-192) | 173.43 ± 5.27 | 174 (160-183) | 172.5 ± 6.98 | 170.5 (164-185) |
| Weight (kg) | 52.5 ± 2.89 *** | 52.5 (50-55) | 66.86 ± 5.80 | 68 (53-80) | 83.04 ± 6.36**** | 82.5 (68-97) | 96.05 ± 9.74**** | 95 (82-114) |
| WC (cm) | 64.75 ± 5.50 **** | 64.5 (60-70) | 83.63 ± 5.59 | 84 (70-101) | 99.65 ± 5.67**** | 100 (87-110) | 107.45 ± 6.90**** | 107.5 (96-124) |
| Hip (cm) | 80 ± 5.77*** | 80 (75-85) | 94.71 ± 4.58 | 96 (80-104) | 104.5 ± 3.56**** | 104.5 (95-110) | 111.2 ± 4.16**** | 111 (104-122) |
| WHR | 0.80 ± 0.01*** | 0.81 (0.8-0.82) | 0.88 ± 0.05 | 0.88 (0.79-1.06) | 0.95 ± 0.05**** | 0.94 (0.87-1.07) | 0.97 ± 0.03**** | 0.97 (0.92-1.03) |
| WHtR | 0.38 ± 0.04**** | 0.37 (0.34-0.42) | 0.49 ± 0.03 | 0.49 (0.39-0.61) | 0.57 ± 0.03**** | 0.57 (0.50-0.66) | 0.62 ± 0.04**** | 0.62 (0.56-0.72) |

* p < 0.05; ** p < 0.01; *** p < 0.005; **** p< 0.0001

**Supplementary Table 2 Characteristics of the study subjects by Chinese BMI category**

| Characteristic | Underweight (n=4) | | Normal (n=81) | | Overweight (n=52) | | Obese (n=44) | |
| --- | --- | --- | --- | --- | --- | --- | --- | --- |
|  | Mean ± SD | Median (range) | Mean ± SD | Median (range) | Mean ± SD | Median (range) | Mean ± SD | Median (range) |
| Age (years) | 27.00 ± 2.16 | 27.5 (24-29) | 30.26 ± 4.22 | 30 (22-49) | 32.27 ± 5.53* | 31 (22-49) | 30.91 ± 4.97 | 31 (21-44) |
| Height (cm) | 172.5 ± 4.93 | 172.5 (167-178) | 173 ± 5.11 | 173 (160-192) | 171.8 ± 5.34 | 171.5 (160-183) | 173.3 ± 5.9 | 173 (161-185) |
| Weight (kg) | 52.50 ± 2.89*** | 52.5 (50-55) | 65.69 ± 5.69 | 66 (53-75) | 76.19 ± 6.29**** | 75.5 (65-90) | 91.11 ± 8.92**** | 90 (75-114) |
| WC (cm) | 64.75 ± 5.50**** | 64.5 (60-70) | 82.64 ± 5.33 | 83 (70-92) | 92.58 ± 6.55**** | 91.5 (82-105) | 105.4 ± 5.93**** | 103.5 (95-124) |
| Hip (cm) | 80.00 ± 5.77*** | 80 (75-85) | 94.28 ± 4.84 | 96 (80-104) | 100.4 ± 4.92**** | 101 (91-110) | 108.3 ± 4.4**** | 108 (100-122) |
| WHR | 0.81 ± 0.01*** | 0.81 (0.80-0.82) | 0.88 ± 0.04 | 0.88 (0.79-0.95) | 0.92 ± 0.05**** | 0.92 (0.81-1.06) | 0.97 ± 0.04**** | 0.97 (0.91-1.07) |
| WHtR | 0.38 ± 0.04*** | 0.37 (0.34-0.42) | 0.48 ± 0.03 | 0.48 (0.39-0.53) | 0.54 ± 0.04**** | 0.54 (0.47-0.61) | 0.61 ± 0.04**** | 0.60 (0.54-0.72) |

* p < 0.05; ** p < 0.01; *** p < 0.005; **** p< 0.0001

**Supplementary Table 3 Characteristics of semen quality parameters, serum reproductive hormones, lipids and leptin by Chinese BMI category**

| Characteristic | Underweight (n=4) | | Normal (n=81) | | Overweight (n=52) | | Obese (n=44) | |
| --- | --- | --- | --- | --- | --- | --- | --- | --- |
|  | Mean ± SD | Median (range) | Mean ± SD | Median (range) | Mean ± SD | Median (range) | Mean ± SD | Median (range) |
| Semen volume (mL) | 2.53 ± 0.33 | 2.4 (2.3-3.0) | 2.4 ± 0.58 | 2.4 (0.5-3.5) | 2.28 ± 0.63 | 2.3 (0.6-4.0) | 2.35 ± 0.67 | 2.45 (0.60-4.00) |
| Ratio of PR sperm (%) | 21.15 ± 6.55 | 22.05 (12.4-28.1) | 37.79 ± 18.61 | 39.6 (0.1-71.4) | 40.64 ± 16.78 | 38.6 (4.1-71.8) | 32.76 ± 21.56 | 29.05 (0-80.60) |
| Ratio of PR + NP sperm (%) | 41.15 ± 8.97 | 44.35 (28.00-47.90) | 57.94 ± 19.80 | 60.2 (7.6-92) | 59.26 ± 18.09 | 62.6 (13.2-89.7) | 52.78 ± 22.96 | 51.7 (6.3-96.0) |
| Sperm concentration (10^6^/ml) | 67.12 ± 38.59 | 73.25 (22.60-99.40) | 49.18 ± 27.24 | 45.9 (3.6-118.4) | 48.72 ± 26.91 | 42.75 (4.2-118.4) | 42.53 ± 29.40 | 40.1 (4.5-107.2) |
| **Ratio of morphologically normal sperm (%)** | 4.05 ± 1.34 | 4.15 (2.5-5.4) | 3.95 ± 2.09 | 4.2 (0.5-14.1) | 3.34 ± 1.64* | 2.9 (0.5-7.4) | 2.95 ± 1.68*** | 2.8 (0.5-7.4) |
| FSH | 3.98 ± 1.53 | 3.97 (2.44-5.54) | 4.40 ± 2.29 | 3.84 (1.31-17.08) | 4.37 ± 1.93 | 3.93 (1.70-10.19) | 5.00 ± 2.28 | 4.54 (2.19-10.75) |
| LH | 3.13 ± 1.22 | 3.26 (1.6-4.0) | 3.51 ± 1.79 | 3.02 (0.9-9.7) | 3.16 ± 1.61 | 2.71 (1.19-7.54) | 3.41 ± 1.21 | 3.39 (0.70-6.46) |
| **P** | 0.69 ± 0.18 | 0.73 (0.44-0.84) | 0.71 ± 0.34 | 0.74 (0.03-1.58) | 0.53 ± 0.25*** | 0.5 (0.17-1.74) | 0.64 ± 0.30 | 0.62 (0.16-1.37) |
| **E2** | 33.75 ± 10.69 | 32 (23-48) | 33.59 ± 13.19 | 32 (12-79) | 36.32 ± 12.71 | 36 (15-69) | 44.09 ± 13.34**** | 46 (20-75) |
| **PRL** | 10.52 ± 3.66 | 9.2 (7.93-15.76) | 9.98 ± 4.95 | 8.74 (3.29-36.58) | 8.44 ± 3.67* | 7.64 (3.29-20.56) | 9.01 ± 3.10 | 9.16 (2.48-16.43) |
| **TT** | 5.32 ± 2.47 | 4.74 (3.08-8.71) | 3.98 ± 1.04 | 3.92 (1.86-6.18) | 3.36 ± 0.84**** | 3.3 (1.84-5.31) | 3.28 ± 1.00**** | 3.21 (1.29-5.48) |
| **E2/TT** | 7.21 ± 3.55 | 6.37 (4.02-12.09) | 9.12 ± 4.71 | 7.99 (2.37-29.33) | 11.55 ± 5.17*** | 10.18 (3.99-27.96) | 14.90 ± 13.34**** | 13.83 (5.01-44.96) |
| **Serum leptin (μg/L)** | 11.55 ± 1.14 | 11.53 (10.32-12.83) | 12.30 ± 2.10 | 12.34 (7.63-17.64) | 14.12 ± 1.66**** | 14.21 (10.13-16.98) | 14.99 ± 2.26**** | 15.62 (8.72-17.88) |
| **Seminal leptin (μg/L)** | 12.85 ± 1.60 | 12.78 (11.14-14.71) | 12.56 ± 2.03 | 12.21 (8.98-18.23) | 15.17 ± 1.98**** | 15.32 (9.6-18.01) | 15.31 ± 2.46**** | 15.56 (8.94-18.80) |
| Seminal SOD (pg/ml) | 214.8 ± 51.38 | 204.4 (166.7-283.7) | 204.7 ± 37.5 | 206.3 (137.8-277.3) | 216.6 ± 36.32 | 219.1 (141.6-281.9) | 214.9 ± 41.24 | 228.7 (143.2-266.3) |
| **TC (mmol/L)** | 4.28 ± 0.59 | 4.1 (3.80-5.12) | 4.54 ± 0.84 | 4.58 (2.71-6.48) | 4.85 ± 0.90 | 4.87 (3.09-7.61) | 5.68 ± 1.30**** | 5.29 (3.35-9.11) |
| **LDL-C (mmol/L)** | 2.44 ± 0.70 | 2.23 (1.90-3.42) | 2.56 ± 0.65 | 2.43 (1.23-4.31) | 2.71 ± 0.73 | 2.83 (1.21-4.12) | 3.55 ± 1.25**** | 3.10 (1.52-5.49) |
| **HDL-C (mmol/L)** | 1.43 ± 0.09* | 1.46 (1.30-1.52) | 1.16 ± 0.23 | 1.12 (0.73-1.71) | 1.09 ± 0.23 | 1.10 (0.64-1.58) | 1.05 ± 0.37 | 1.10 (0.17-1.82) |
| **TG (mmol/L)** | 0.89 ± 0.32* | 0.85 (0.55-1.32) | 1.93 ± 1.48 | 1.74 (0.26-13.14) | 2.49 ± 1.32** | 2.20 (0.91-5.85) | 3.04 ± 1.37**** | 2.71 (0.83-5.62) |

* p < 0.05; ** p < 0.01; *** p < 0.005; **** p< 0.0001

**Supplementary Table 4 Univariate and multivariate analysis of semen parameters, serum reproductive hormone, lipids and leptin levels regarding Chinese BMI category (normal: 18.50–23.9 versus pathologic >23.9)**

| Variable | Univariate | | Multivariate Model A | | Multivariate Model B | | Multivariate Model C | |
| --- | --- | --- | --- | --- | --- | --- | --- | --- |
|  | RR (95% CI) | P value | RR (95% CI) | P value | RR (95% CI) | P value | RR (95% CI) | P value |
| Semen volume (mL) | 1.9 (0.94-3.9) | 0.072 | 1.60 (0.77 - 3.3) | 0.205 | - | - | 1.00 (0.379 - 2.7) | 0.994 |
| Ratio of PR sperm (%) | 1.5 (0.97-2.2) | 0.066 | 1.58 (0.91 - 2.7) | 0.101 | - | - | 1.67 (0.855 - 3.3) | 0.133 |
| Ratio of PR + NP sperm (%) | 1.1 (0.69-1.8) | 0.660 | 0.61 (0.29 - 1.3) | 0.207 | - | - | 0.59 (0.259 - 1.3) | 0.207 |
| Sperm concentration (10^6^/ml) | 1.4 (0.78-2.5) | 0.260 | 1.50 (0.70 - 3.2) | 0.296 | - | - | 2.03 (0.838 - 4.9) | 0.117 |
| Ratio of morphologically normal sperm (%) | 0.96 (0.64-1.4) | 0.850 | 0.99 (0.66 - 1.5) | 0.968 | - | - | 1.09 (0.648 - 1.8) | 0.754 |
| E2 | 1.2 (0.57-2.5) | 0.630 | - | - | 0.51 (0.219 - 1.2) | 0.112 | 0.43 (0.168 - 1.1) | 0.073 |
| PRL | 0.96 (0.54-1.7) | 0.890 | - | - | 1.14 (0.591 - 2.2) | 0.698 | 0.98 (0.482 - 2.0) | 0.956 |
| Serum leptin (μg/L) | 1.2 (0.78-1.9) | 0.380 | - | - | 0.91 (0.503 - 1.6) | 0.749 | 0.79 (0.386 - 1.6) | 0.514 |
| Seminal leptin (μg/L) | 1.1 (0.69-1.7) | 0.720 | - | - | 1.33 (0.739 - 2.4) | 0.339 | 1.51 (0.797 - 2.9) | 0.205 |
| **TC (mmol/L)** |  | **0.007** |  |  |  |  |  |  |
| < 5.18 | 1.00 |  | - | - | 1.00 |  | 1.00 |  |
| **≥ 5.18 & ≤ 6.19** | 0.33 (1.2-3.5) |  | - | - | 2.12 (0.940 - 4.8) | 0.070 | 2.51 (1.050 - 6.0) | **0.039** |
| > 6.2 | -0.33 (0.38-1.4) |  | - | - | 0.32 (0.072 - 1.4) | 0.125 | 0.33 (0.075 - 1.5) | 0.145 |
| LDL-C (mmol/L) |  | 0.630 |  |  |  |  |  |  |
| <3.37 | 1.00 |  | - | - | 1.00 |  | 1.00 |  |
| ≥3.37 & ≤ 4.12 | -0.16 (0.71-2.3) |  | - | - | 0.98 (0.411 - 2.4) | 0.970 | 0.66 (0.257 - 1.7) | 0.394 |
| >4.14 | -0.16 (0.42-1.7) |  | - | - | 2.55 (0.583 - 11.1) | 0.214 | 2.31 (0.570 - 9.4) | 0.241 |
| HDL-C (mmol/L) |  | 0.910 |  |  |  |  |  |  |
| <1.04 | 1.00 |  | - | - | 1.00 |  | 1.00 |  |
| ≥1.04 & <1.55 | 0.2 (0.69-1.7) |  | - | - | 1.22 (0.735 - 2.0) | 0.442 | 1.22 (0.704 - 2.1) | 0.473 |
| ≥1.55 | 0.2 (0.43-3.5) |  | - | - | 1.19 (0.372 - 3.8) | 0.768 | 1.06 (0.310 - 3.6) | 0.927 |
| TG (mmol/L) | 0.87 (0.56-1.3) | 0.510 | - | - | 0.81 (0.496 - 1.3) | 0.381 | 0.90 (0.526 - 1.5) | 0.700 |

**Supplementary Table 5 Association between sperm concentration and obesity-associated markers**

|  | Oligospermia | | Normospermia | | P value |
| --- | --- | --- | --- | --- | --- |
|  | N | RR (95% CI) | N | RR (95% CI) |  |
| WHO BMI category |  |  |  |  | 0.093 |
| Underweight | 0 | 0.38 (0.23-1.8) | 4 | 0 (referent) |  |
| Normal | 7 | 1.00 | 96 | 0 (referent) |  |
| Overweight | 9 | 0.38 (1-2.1) | 45 | 0 (referent) |  |
| Obese | 3 | 0.38 (0.5-1.4) | 17 | 0 (referent) |  |
| Chinese BMI category |  |  |  |  | 0.800 |
| Underweight | 0 | 0.029 (0.23-1.7) | 4 | 0 (referent) |  |
| Normal | 6 | 1.00 | 75 | 0 (referent) |  |
| Overweight | 4 | 0.029 (0.72-1.5) | 48 | 0 (referent) |  |
| Obese | 9 | 0.029 (0.7-1.6) | 35 | 0 (referent) |  |
| WC (cm) |  |  |  |  | 0.210 |
| < 90 | 6 | 1.2 (0.9-1.7) | 13 | 0 (referent) |  |
| ≥ 90 | 89 | 1.00 | 74 | 0 (referent) |  |
| WHR |  |  |  |  |  |
| < 90 | 3 | 0.93 (0.68-1.3) | 16 | 0 (referent) | 0.670 |
| ≥ 90 | 73 | 1.00 | 89 | 0 (referent) |  |
| WHtR |  |  |  |  |  |
| <0.5 | 4 | 1.1 (0.84-1.6) | 15 | 0 (referent) | 0.400 |
| ≥0.5 | 66 | 1.00 | 96 | 0 (referent) |  |
